# Mapping of care pathways in pediatric and adult palliative care in Spain: A case study

**DOI:** 10.1017/S1478951525000513

**Published:** 2025-05-23

**Authors:** Tania Ruiz-Gil, Francisco Ródenas-Rigla, Zoe Valero-Ramon, María Dolores Rodríguez Rabadán

**Affiliations:** 1 Polibienestar Research Institute, Universitat de València, Valencia, Spain; 2 ITACA-SABIEN, Universitat Politècnica de València, Valencia, Spain; 3 Multiprofessional Pediatric Teaching Unit of the Region of Murcia (UDMP-RM), Hospital Clínico Universitario Virgen de la Arrixaca, El Palmar, Murcia, Spain

**Keywords:** Palliative care, Pediatric palliative care, Process mining, Care pathways, Patient-centered care, Home care, Healthcare process optimization

## Abstract

**Objectives:**

This study aimed to map the actual care pathways for pediatric and adult palliative care (PC) patients at a hospital in the Region of Murcia (Spain) utilizing Process Mining (PM) techniques. The goal was to identify inefficiencies and areas for improvement in providing comprehensive and coordinated care to enhance patient outcomes.

**Methods:**

A retrospective review of anonymized clinical records was conducted, covering data from 2002 to 2021 for adult patients and from 2001 to 2021 for pediatric patients. The final dataset for adults comprised records from 85 patients and 2,696 episodes, and, for pediatric patients, the dataset included 57 individuals with 1,912 episodes. PM techniques (concretely, PMApp) facilitated the visualization and evaluation of actual care pathways, compared to theoretical models, highlighting bottlenecks and variabilities.

**Results:**

The analysis revealed distinct care pathways for adult and pediatric patients. Pediatric pathways showed inconsistencies with theoretical models due to variability in diseases and care needs, while adult pathways aligned better with expectations. Key inefficiencies included delays in shifting to home care and multiple visits to the hospital Emergency Department before referral to specialized teams. Simplified process models provided clearer insights into frequent care pathways and highlighted critical transition points, supporting optimization strategies.

**Significance of results:**

The findings underscore the utility of PM in enhancing care pathway transparency, identifying inefficiencies, and supporting data-driven process redesign. The study advocates for updating theoretical models and adopting structured data collection to reduce variability and improve PC delivery. These measures are critical for achieving consistent, patient-centered care across diverse healthcare settings.

## Introduction

Palliative care (PC) has significantly advanced globally, driven by the World Health Organization and supported by experiences in pioneering countries like the United Kingdom, which introduced community-based hospices as a model of care, and Canada, where home-based PC predominates, prioritizing integrated and home-based care (Calvo Llorca 2019). Recognizing its necessity, World Health Assembly Resolution 67.19 in 2014 urged PC integration into healthcare systems for supporting patients with advanced chronic or terminal illnesses at all life stages (WHO 2021). Internationally, pediatric PC has emerged as a specialized area due to the variety of ages, conditions, and unique needs of pediatric patients, from prenatal stages to young adulthood (Lohman et al. 2022). This approach includes care adapted to children’s physical, emotional and cognitive development, emphasizing family’s central role in care and decision-making (Roland et al. 2022).

In Spain, PC development has been gradual, resulting in national policies such as the “Palliative Care Strategy of the National Healthcare System (2010–2014 Update),” and the document “Paediatric Palliative Care in the National Healthcare System: Criteria for Care” (Ministerio de Sanidad, Servicios Sociales e Igualdad 2014). However, these documents require updating, as a decade has passed since their formulation. Nonetheless, in pediatric PC, the “Clinical Practice Guideline on Palliative Care in Paediatrics” was published in 2022 (Ministerio de Sanidad nd).

Regarding the scope of this research, the development of PC in the Region of Murcia (Spain), has progressed significantly since its implementation for pediatric and adult patients. The process began in 2007 with the creation of the “Comprehensive Palliative Care Plan,” following the National PC Strategy guidelines (Servicio Murciano de Salud 2007). In parallel, pediatric PC was further consolidated in 2013 with the creation of the Regional Unit for Home Hospitalization and Pediatric Palliative Care (URHD-CPP, in Spanish), integrating highly specialized resources for home-based care for children with palliative needs and chronic illnesses (Servicio Murciano de Salud 2024).

By 2021, adult patient teams served over 3,000 patients annually across the region; while pediatric teams, managed about 180 cases, consolidated their multidisciplinary work and collaborating with associations to support children and families’ well-being (Servicio Murciano de Salud 2024).

Despite these advances, the lack of a unified PC circuit and definitive coding for the care process present challenges for organizing care pathways within referral hospitals like the University Clinical Hospital Virgen de la Arrixaca of the Murcian Healthcare Service. This hospital, a reference for Health Area 1 (Murcia-West), is part of the 9 available areas and lacks a specific PC structural strategy. This variability both at the healthcare center and regional level has led to heterogeneous management of PC in each health area. This results in the absence of a homogeneous pathway within the hospital, affecting pediatric and adult patients. Given this situation, we identify Process Mining (PM), defined by van der Aalst (2016), as the ideal tool. It is a relatively new research discipline focused on extracting knowledge from data generated by processes stored in databases, known as event logs. These event logs are detailed records of activities within a system, capturing crucial information about who performed what action, when, and in what context. PM offers tools, algorithms, and visualization techniques that enable experts to gain insights into the characteristics of process execution. PM provides a process-oriented perspective on performance and behavior by analyzing event logs on a specific procedure.

In healthcare, these processes consist of structured multidisciplinary care protocols outlining steps for managing patients with specific clinical issues (Campbell et al. 1998). In this regard, care pathways represent complex processes encompassing every stage of managing a patient’s condition over a specified timeframe, including details on progress and outcomes.

Although PM use in healthcare is emergent, studies have demonstrated its feasibility (Rojas et al. 2016). PM techniques have been used for administrative analysis in healthcare domains for specific illnesses (Yoo et al. 2016), like Gynecologic Oncology (Mans et al. 2008), or for the analysis of services like Emergency Departments (Ibanez-Sanchez et al. 2019).

Despite these contributions, further research is currently needed to explore incorporating PM techniques to analyze care pathways in PC for pediatric and adult patients.

In this context, this study aims to map, utilizing PM techniques, the actual care pathway in PC for pediatric and adult patients at the University Clinical Hospital Virgen de la Arrixaca (Region of Murcia, Spain), specifically. To this end, existing data will be used to identify possible areas for improvement and optimization in the comprehensive care provided to patients and their families.

## Methods

### Case study: PC at the university clinical hospital Virgen de la Arrixaca

This section describes the theoretical model used in the hospital for pediatric and adult palliative patients. For pediatric patients, their access to the URHD-CPP, the central unit of the theoretical model, begins with a request for assistance from the URHD-CPP, other pediatric services at the University Clinical Hospital Virgen de la Arrixaca, or other hospitals with pediatric care. Once the request is submitted, the next step is a referral request through the SELENE program, where a specialized team evaluates eligibility for PC based on inclusion criteria. SELENE is a unique computerized system for patient care management, integrating Primary, Specialized, and Mental Healthcare, as well as diagnostic imaging, laboratory and scheduling functions. This process prioritizes patients in end-of-life situations, those with acute exacerbations of complex chronic conditions requiring home care, and those with high social needs.

After evaluation, a decision is made regarding hospital admission or home care, with an initial visit in both cases. The level of care required and the necessary palliative follow-up processes are also established. There are 3 levels of PC for minors (from lower to higher complexity), with the URHD-CPP responsible for patients with the highest complexity (level 3), coordinating with primary care pediatricians and other specialists. Minors at levels 1 and 2 are assessed by the Unit, establishing a care plan that can be delegated to hospital or primary care pediatricians as appropriate.

**Figure 1.**
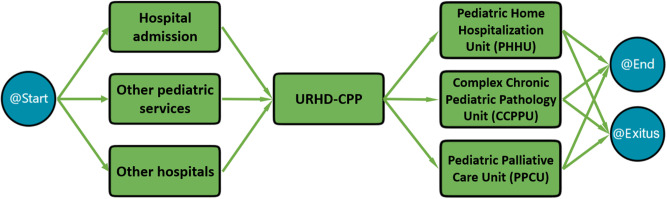
Theoretical model of the palliative care process for pediatric patients in the university clinical hospital Virgen de la Arrixaca.

At the end-of-life stage, the PC healthcare team (including social workers, psychologists, associations and volunteers) provides telephone support or home visits, offering emotional and practical support to patients and families. If a patient visits the Emergency Department, an alert in the SELENE system will indicate that they are a complex chronic or PC patient, with their clinical record summarizing multidisciplinary care, key diagnoses, devices in use, chronic pharmacological treatments, actions that should (or should not) be carried out, etc. Upon the patient’s death, a protocol activates grief support from the multidisciplinary team, including visits and phone contact within a month or as needed. The entire process described is shown in Fig. 1.

For adult patients, the hospital has a Palliative Care Support Team operating across various care levels in both home and hospital settings through the Home Care Support Team (HCST) and the Hospital Care Support Team. In Health Area 1 (Murcia-West), patients access specialized teams according to their pathologies, either through the Oncology Department or the Hospital Care Support Team, which are responsible for coordinating referrals to PC.

**Figure 2.**
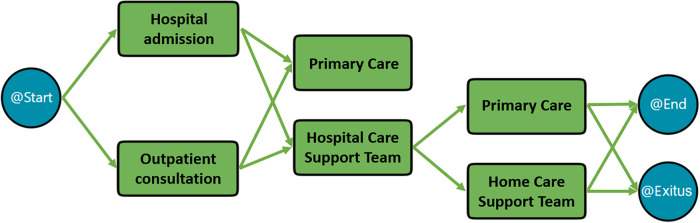
Theoretical model of the palliative care process for adult patients in the university clinical hospital Virgen de la Arrixaca.

Source: own elaboration.

The access pathway for adult patients begins when they are admitted to the hospital or attend an outpatient consultation. At this point, the referring specialist performs a multidimensional assessment and identifies whether the patient has PC needs and initiates the request for assistance. Depending on the situation, the patient may be referred to Primary Care or the Hospital Care Support Team for further evaluation. If home follow-up is required, the HCST contacts the patient and/or family within 48 h to assess the level of complexity and coordinates with Primary Care team for the follow-up. This sequence of steps, as described in the theoretical model, is visually represented in Fig. 2.

### Sample and data collection procedure

This retrospective study was approved by the Ethics Committee of the University Clinical Hospital Virgen de la Arrixaca (ref. 2020-9-3). Patients had to be coded as having palliative needs or chronic clinical complexity, and had already been referred to PC services to be included. An anonymized review of clinical records was conducted for adult patients from 2002 to 2021 and pediatric patients from 2001 to 2021. Finally, the adult patient dataset included information from 85 patients with 2,747 episodes; after removing incomplete episodes, 2,696 episodes were analyzed. The pediatric dataset included 57 patients with 1,912 episodes, all analyzed.

Data collection followed a rigorous protocol to ensure information accuracy and integrity. Electronic clinical record management systems from the hospital were used to access data. The collected information included admission reports, discharge summaries, progression notes, outpatient consultation reports, treatment details, and records from various services and units. These records were in plain text and contained detailed information on patient follow-ups and clinical summaries.

After data collection, a structured database was created using Microsoft Excel to manage the information. Four research team members participated in this process, with a background in Social Sciences (Sociology and Social Work). The database was organized into 2 tables, one for adult patients and another for pediatric patients, including data on each episode documented in the reports. Two research team members extracted relevant information through an in-depth reading of all reports, identifying data necessary for populating the tables and manually entering information to avoid duplicate data across multiple reports. Finally, 4 research team members reviewed the entire process.

Each patient was assigned a unique code to ensure confidentiality, without any personal data connection. Each recorded episode represented a specific status or stage in the care process, identifying the responsible service or unit. The database included the start dates, indicating the time the report was recorded and thus the date of the care event; and the end dates of each episode, which, when not explicitly stated, were inferred from the information in the reports or assumed as the start date of the subsequent event. Laboratory test dates, imaging, and other procedures performed during each episode, along with associated referrals, were also recorded.

Erroneous or incomplete date episodes in the adult patient database were removed, and episodes from similar units or services (e.g., “Outpatient” “Advanced Outpatient,” “Outpatient Review for Palliative Care”) were grouped under a unified label (“Outpatient”). The responsible service for each episode was recorded. To protect sensitive data, the records were anonymized, and data protection measures were implemented in compliance with the General Data Protection Regulation and current data protection legislation. The database will be available for consultation upon request.

### Data analysis

As described, we used PM techniques to analyze the data; concretely, we utilized PMApp (Ibanez-Sanchez et al. 2023) as a PM tool. PMApp, the Interactive PM Toolkit, is a specialized tool for healthcare professionals. It supports the visualization of real-world data from healthcare organizations and enables the co-creation of advanced process views, thereby enriching the analyses conducted by healthcare stakeholders (Ibanez-Sanchez et al. 2019).

Data analysis was performed in several phases. It started with data ingestion (from the database), where the Data Log was obtained to perform the PM. Secondly, data was processed to compute the needed variables to create the events and the traces for the analysis. Concretely, we computed the Patient ID (unique code created for the study), the episode and the start date. The process model behind the data was obtained by applying the PALIA (Parallel Activity Log Inference Algorithm) discovery algorithm incorporated into the PMApp tool (Fernandez-Llatas et al. 2015).

PALIA has been widely tested in real healthcare scenarios. It has been applied to the analysis of follow-up protocols of patients with diabetes (Conca et al. 2018; Concaro et al. 2009), for the characterization of emergency flows, measuring organizational changes effects (Ibanez-Sanchez et al. 2019), for discovering Surgery Department flow (Fernandez-Llatas et al. 2015), or for characterizing the prostate cancer care unit (Valero-Ramon et al. 2023).

After the discovery phase, the metadata associated with the model was computed. PMApp supports various forms of metadata related to models, including statistical information about nodes and transitions and the relationships between the model’s topological structures and log events. In PMApp, users can create visualizations that enhance the discovered model through color gradients. This feature allows for the generation of specific maps that highlight situations based on customized formulations represented by nodes. Such techniques facilitate healthcare professionals’ understanding of processes. Maps can be designed for standard metrics, including performance, activity duration, case counts, and event numbers. The result was 2 graphical models of the care processes for pediatric and adult patients needing PC at University Clinical Hospital Virgen de la Arrixaca.

## Results

As stated in previous sections, we demonstrated the possibilities of applying PM techniques to discover the care pathways in PC for pediatric and adult patients at the considered hospital. The results were 2 care pathway models.

The figures in this study depict 2 complementary views of the care workflows obtained using PM techniques: the complete workflow and the most frequent care episodes. The complete workflow represents all care episodes, including both frequent and infrequent ones, providing a holistic view of the process. In contrast, the figures focusing on the most frequent care episodes highlight the 80% most common pathways while hiding the less frequent episodes, which are represented in lighter colors in the complete workflow. This distinction is essential, as the figures with the most frequent episodes emphasize the parts of the process most relevant to healthcare professionals, enabling them to focus on key patterns while deprioritizing rare or exceptional cases. These visualizations, included in the supplementary material, provide a clearer understanding of the actual patient pathways.

### Pediatrics care process

**Figure 3.**
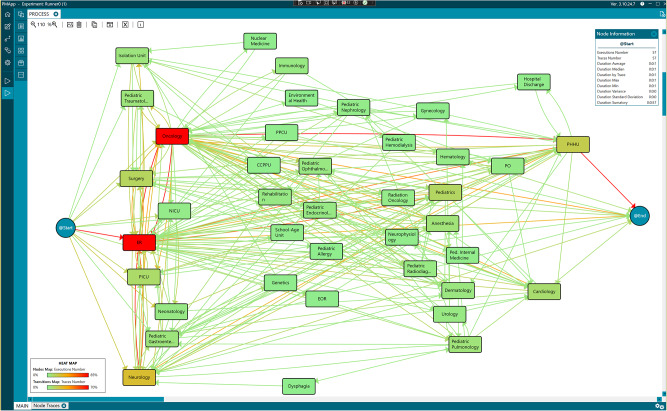
Workflow of the pediatric care process obtained by process mining.

After analyzing the pediatric dataset, the actual care flow followed by the pediatric patients was discovered. Figure 3 graphically illustrates the generated model that depicts the sequence of events and the transition between them.

The model illustrates the care process based on the analyzed data, where each node represents an event or episode in the process, such as “ER” (Emergency Room episode). The arrows between nodes indicate transitions between episodes, reflecting the flow of patients through the care process. Artificial nodes, @Start and @End, were added to mark the beginning and end of the process. This does not imply that the overall process starts or ends at these points, but rather that the data available starts and ends then.

To simplify the analysis, consecutive identical episodes were grouped into a single event, assuming that the end of one episode coincides with the start of the next. This temporal sequencing provides an abstract view of the care process followed by the patients.

To improve interpretability, a heatmap was applied to the nodes and transitions in the figures. A greater intensity of red within the nodes indicates a longer duration of stay in that episode, while green represents shorter stays. Similarly, a more intense red in the transitions signifies a higher number of cases (patients) associated with that transition, as outlined in the heatmap legend incorporated into each relevant figure to ensure clarity and consistency. This visualization supports identifying bottlenecks or critical points in the care process, where patients spend more time or transit in higher numbers, offering a comprehensive and dynamic perspective of the actual patient care flow.

**Figure 4.**
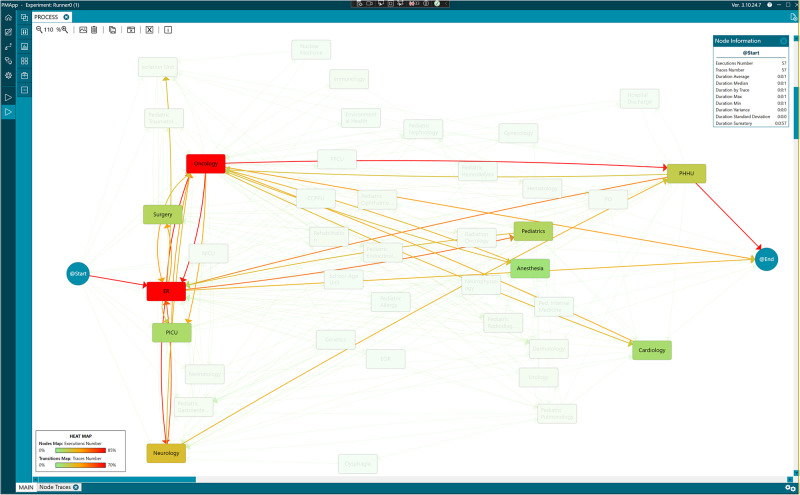
The most frequent pathway in the care process for pediatric patients.

Source: Generated with PMApp tool.

The analysis also focused on identifying the most common pathways followed by the patients. An abstraction of the process was performed, considering 80% of the most frequent data. In this process view, the number of times each episode occurs is highlighted, while nodes/episodes that occur in less than 20% are hidden. This ensures that the most frequent pathways followed by pediatric patients are emphasized, as shown in Fig. 4.

### Adults care process

**Figure 5.**
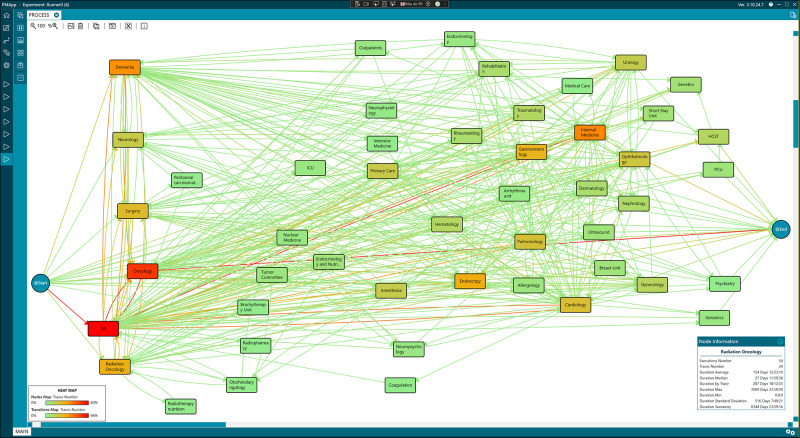
Workflow of the care process for adult patients.

Following the same procedure for the adult patient’s data set and simplifying the data, the actual care process for adult patients was discovered (see Fig. 5). This first model was an intricate workflow representing a highly complex care process followed by the patients, with numerous activities and transitions between them, making it understandability and interpretability difficult.

The model revealed that Emergency and Oncology episodes were the most common in the workflow. To improve the interpretability of the model, Emergency episodes were removed to obtain a new simplified version of the model, which is shown in the supplementary material (see Figure S1).

Still, the new model highlighted the complexity of the actual care process. In a subsequent step, episodes related to Outpatient consultations, such as Cardiology, Dermatology, Endocrinology, Neurology, and others, were grouped under the same episode, Outpatient, to bring the real model closer to the theoretical one. This new version of the model, which identifies the 3 main steps of the process, is presented in the supplementary material (see Figure S2). The initial steps corresponding to the admission are included in the purple rectangle, mainly through Emergency, Outpatient, and Oncology. The blue rectangle groups intermediate steps like surgeries, therapies, and rehabilitation. Lastly, the red rectangle includes the final steps of the process, highlighting the Palliative Care Unit (PCU) and the HCST episodes.

**Figure 6.**
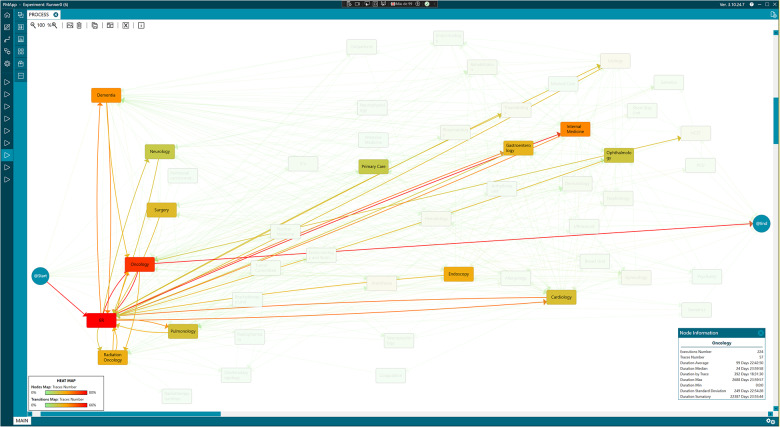
Most frequent care process in adult patients.

Source: Generated with PMApp tool.

Finally, the most frequent care pathways were extracted from the original workflow to enhance the model’s clarity. Figure 6 illustrates the predominant pathway by abstracting the process, presenting 80% of the most common data while omitting pathways that occur in less than 20% of cases. This focused model enables a clear visualization of the primary trajectories within the care process. It reveals that patients typically experience multiple episodes in the ER and Oncology, followed by involvement with Internal Medicine and Dementia care. In contrast, referrals to the HCST and the PCU were notably less frequent and did not rank among the most common episodes.

This information is essential for healthcare professionals, as it highlights the high level of use of Emergency services by these patients, even when they are referred to specific services.

## Discussion

In this study, we employ PM techniques to uncover the actual care pathways for PC, analyzing data from both pediatric and adult patients at the University Clinical Hospital Virgen de la Arrixaca. Furthermore, we take an additional step by analyzing and comparing these actual workflows with the theoretical models provided by the hospital’s healthcare professionals.

When analyzing adult patients’ cases with healthcare professionals from the hospital, differences between the care process observed in practice and the theoretical model become evident, particularly in the intermediate steps of care. In practice, the pathway to accessing PC appears to be more complex than depicted in theoretical models, where these intermediate steps are not represented. Comparing both workflows, the theoretical and the one inferred from the data, helped identify areas for improvement. This analysis revealed that, in clinical practice, the lack of implementation of well-defined pathways can lead to non-optimized care pathways, such as multiple visits to the Emergency Department before referral to home PC. However, it is important to note that once patients are referred to home and PC units, emergency visits decreases, which is logical given that referrals to these units typically occur at the end of the care process. This pattern aligns with other studies highlighting similar bottlenecks in care flow, affecting system efficiency (Sagardía et al. 2019).

Furthermore, according to recent studies such as Ingle et al. (2022), many PC interventions lack a clear theoretical basis, as only 38% of the reviewed clinical trials explicitly mentioned a theoretical framework in their design. This highlights the need to develop and validate specific theoretical frameworks to explain PC dynamics and outcomes, which could improve the alignment between real processes and planned pathways. It is also crucial to adapt the models to the contextual and demographic variations of patients, as seen in Pediatrics and those with chronic diseases (García-Trevijano-Cabetas et al. 2022).

Regarding pediatric patients, comparing the theoretical PC model with the actual workflow discovered using PM techniques revealed inconsistencies. Many care events in the theoretical model were not reflected in the available clinical data, complicating accurate comparison. Additionally, some episodes in the actual care process were unforeseen in the theoretical model, due to the complexity and variability of pediatric diseases, which complicates standardizing care pathways (Navarro Vilarrubí 2018).

These inconsistencies highlight the need for structured frameworks to guide pediatric PC. In this regard, a new comprehensive care model for pediatric patients with complex chronic diseases and PC needs was recently published in the Region of Murcia (Servicio Murciano de Salud 2024). This model provides a structured framework for the care of these patients, facilitating coordination between care levels and improving continuity of care. Comparing the care pathway discovered in this study with the guidelines of this new model will allow us to assess its implementation and identify potential areas for improvement in the actual care provided to these patients.

Incorporating clear theoretical frameworks in interventions design could optimize care pathways, reduce variability in care and improve patient outcomes. As Ingle et al. (2022) highlight, theoretical frameworks provide a structured basis for selecting key process and outcome indicators, strengthening the replicability and rigor of interventions. Additionally, improving the care flow requires a comprehensive redesign of the processes since quality care can only be provided when it is ensured that the right patient receives the appropriate care in the right place, by the right professional, with the relevant information, and at the right time (Sagardía et al. 2019).

In this sense, it is essential to advance towards creating theoretical models adapted to the particularities of PC in pediatric patients and adults, which could facilitate the planning and execution of more coherent and effective pathways.

Despite the findings, this study has limitations that should be considered when interpreting the results. First, it is a case study conducted in a single hospital, limiting the generalizability of the results to other institutions or healthcare areas. Therefore, the conclusions may not represent other settings, as each hospital or region may have different healthcare structures and protocols. Moreover, the lack of a comparative framework with other regions, both nationally and internationally limits contrasting these findings with those from other healthcare systems. The lack of a longer temporal horizon may also have excluded recent changes in PC policies or infrastructure, affecting the relevance or validity of some results.

Another limitation is the data processing and extraction. Clinical information is mainly stored in free text format, making automated analysis difficult. Additionally, the data extraction process involved manual reading and coding, introducing potential errors and biases. This methodology is not scalable and limits the accuracy of mapping the care process. The discovered model, with more episodes than the theoretical one, makes a direct comparison impossible, as many key points of the theoretical model cannot be mapped due to a lack of relevant data in the clinical reports. Therefore, rethinking theoretical protocols and reviewing data collection is critical for a more precise comparison between theoretical and real processes.

Moreover, the data used in this study was exclusively obtained from the SELENE management system, which may not accurately reflect all aspects of the care process, particularly psychosocial interventions and emotional support for families.

Finally, the study does not assess whether the referral to PC services was timely or clinically appropriate. Instead, it provides a descriptive mapping of the actual referral pathways based on available medical records. Future studies could incorporate prospective designs to analyze the clinical adequacy of referrals and their impact on patient outcomes.

## Conclusions

Based on the results obtained, several measures are proposed to improve both the care process and its analysis. First, the theoretical models of PC should be reviewed and updated to better reflect the clinical reality in both Pediatrics and adults.

Second, Second, improving clinical data collection and processing is crucial. The adoption of more structured data formats and the implementation of technological tools for automated analysis would reduce manual processing errors and improve the scalability of the analysis, allowing for a more accurate comparison between theoretical and real processes.

Finally, continuous analysis of daily practice will help to optimize the theoretical models and allow the identification of bottlenecks and areas for improvement in patient care. This approach will contribute to better resource allocation and greater efficiency within the hospital system, ultimately improving the quality of care provided.

## Supporting information

Ruiz-Gil et al. supplementary material 1 Ruiz-Gil et al. supplementary material

Ruiz-Gil et al. supplementary material 2 Ruiz-Gil et al. supplementary material
