# Supplementary figures and images for: Mapping of care pathways in pediatric and adult palliative care in Spain: A case study
Source: Palliat Support Care. 2025 May 23;23:e107. doi: 10.1017/S1478951525000513 (PMC13166610; doi:10.1017/S1478951525000513)

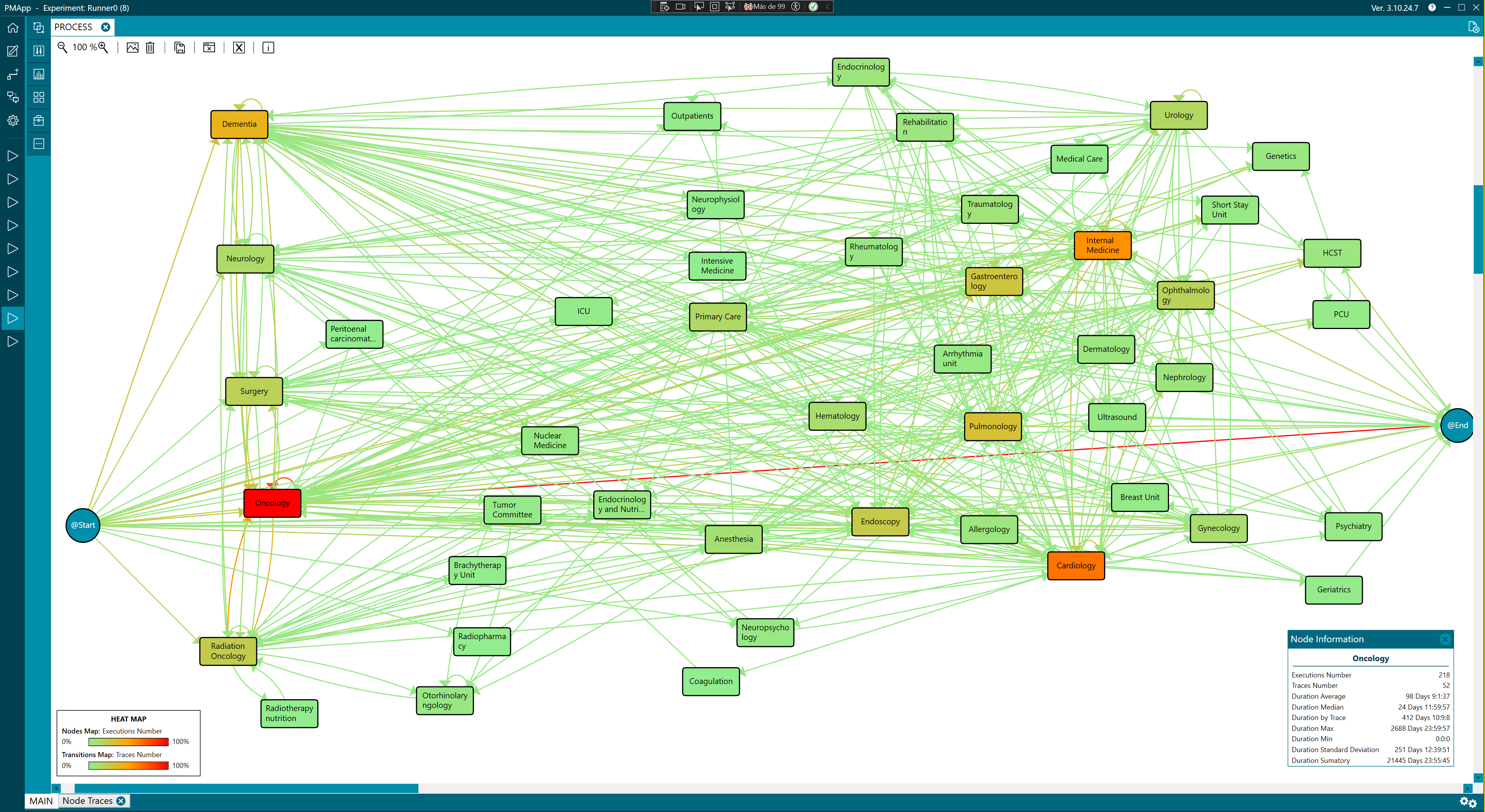

Supplement: Ruiz-Gil et al. supplementary material 1 — Ruiz-Gil et al. supplementary material [file S1478951525000513sup001.png]

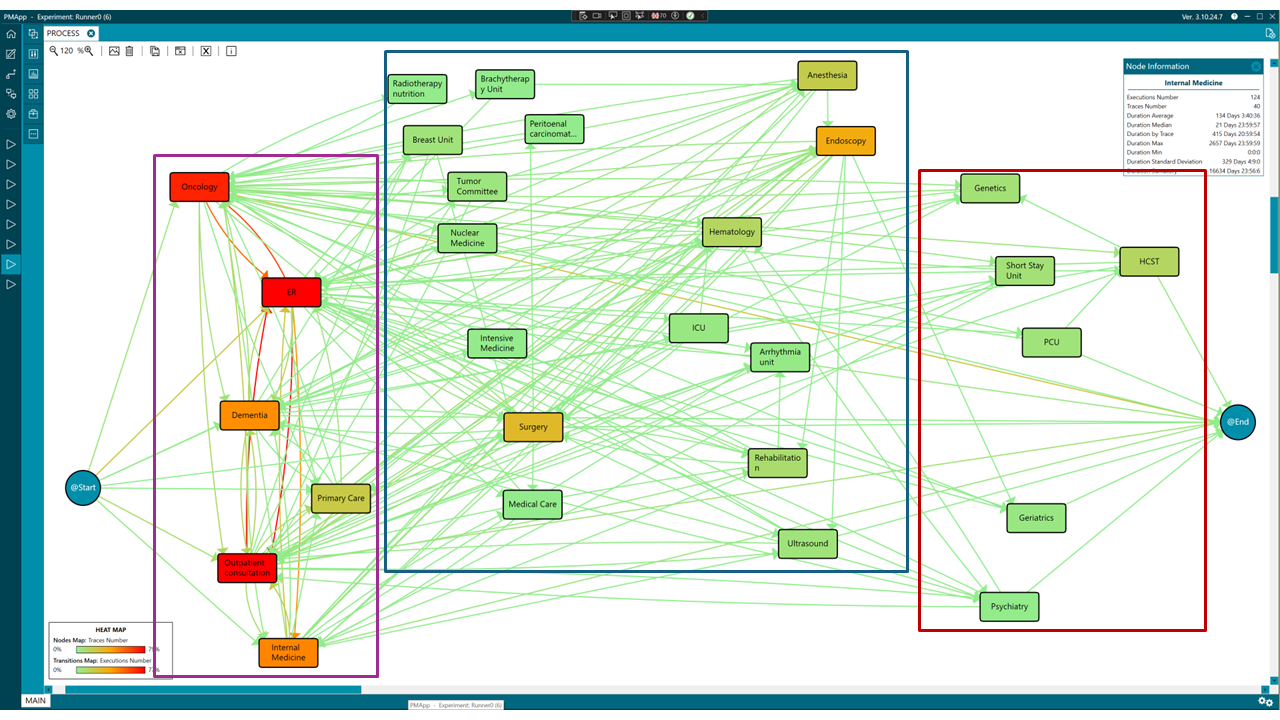

Supplement: Ruiz-Gil et al. supplementary material 2 — Ruiz-Gil et al. supplementary material [file S1478951525000513sup002.png]
